# The trickle before the torrent—diffraction data from X-ray lasers

**DOI:** 10.1038/sdata.2016.59

**Published:** 2016-08-01

**Authors:** Filipe R.N.C. Maia, Janos Hajdu

**Affiliations:** 1 Department of Cell and Molecular Biology, Laboratory of Molecular Biophysics, Uppsala University, Husargatan 3 (Box 596), SE-751 24 Uppsala, Sweden

**Keywords:** X-rays, Structural biology, Imaging

## Abstract

Today Scientific Data launched a collection of publications describing data from X-ray free-electron lasers under the theme ‘Structural Biology Applications of X-ray Lasers’. The papers cover data on nanocrystals, single virus particles, isolated cell organelles, and living cells. All data are deposited with the Coherent X-ray Imaging Data Bank (CXIDB) and available to the scientific community to develop ideas, tools and procedures to meet challenges with the expected torrents of data from new X-ray lasers, capable of producing billion exposures per day.

## Comment

The Protein Data Bank (PDB)^1^ has accumulated more than one hundred thousand structures over a period of nearly 50 years, and on 23 February 2016, it became a billion-atom archive. Each of the structures in the PDB required the collection of more than one X-ray data set, representing a few thousand individual diffraction patterns. One may estimate that a grand total of about a billion diffraction patterns were used so far for determining structures deposited in the PDB. This took nearly 50 years.

The European X-ray Free-Electron Laser (XFEL)^2^ offers the possibility to record a billion diffraction patterns *in a single working day* on objects as small as single macromolecules or as big as nanocrystals and living cells. The opportunities ahead are extraordinary and so are the challenges in data handling and data management. The European XFEL will start user operations in 2017. An upgraded version of the Linac Coherent Light Source^3^ will reach similar operational parameters within a few years.

There is a need for new approaches in sample preparation, sample delivery, data capture, data analysis and interpretation. The Collection of Data Descriptors launched today at *Scientific Data* will help this process and heralds the beginning of an explosive new era^4–9^ (http://www.nature.com/sdata/collections/xfel-biodata).

The six data descriptors in the Collection come from the Linac Coherent Light Source (LCLS)^3^, the first hard X-ray FEL in the world. LCLS started user operations in 2009 and quickly produced the first structural data on biological samples^10,11^. LCLS delivers just over 10 million X-ray pulses per day and the peak brightness of these pulses exceeds that of present synchrotrons by 10^10^. The coherence degeneracy parameters exceed conventional synchrotrons by 10^9^, and the time resolution that can be achieved is nearly 10^5^ times better. LCLS represents a big leap forward. Theory predicts that with an ultra-short and very bright coherent X-ray pulse, a single diffraction pattern may be recorded from a large macromolecule, a virus, or a cell before the sample explodes and turns into a plasma. The over-sampled diffraction pattern permits phase retrieval and hence structure determination^12–15^.

**Figure 1:**
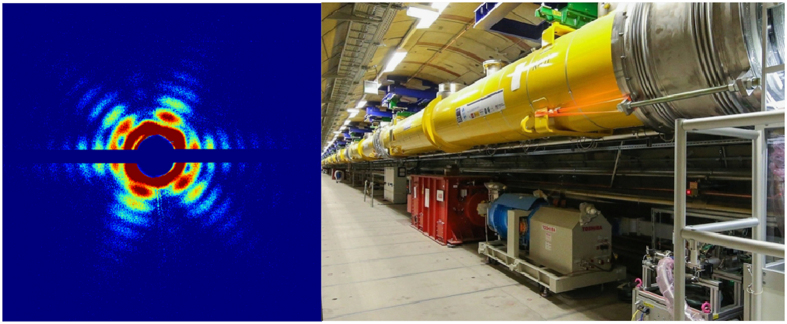
Diffraction patterns of a Mimivirus obtained at the LCLS^5^, and the newly built accelerator at the European XFEL (image on the right, courtesy of the European XFEL). The Collection of Data Descriptors contains many terabytes of images, but they will be quickly dwarfed by the data production rates of upcoming facilities such as the European XFEL and the upgraded LCLS II.

The data described in this Collection exploit the phenomenon of ‘diffraction before destruction’^16,17^. The papers present data on nanocrystals of membrane proteins^4,9^, on single virus particles^5,8^, on isolated cell organelles^6^, and on single living cells^7^, and represent some of the first structural results from the LCLS (Fig. 1).

### The Data Challenge

The development of fast detector systems in recent years has been driven by the wish to match the great advances in X-ray sources and by the desire to capture structural dynamics with high temporal and spatial resolution. The resulting increase in the volume of data has thrown X-ray imaging scientists into the midst of the Big Data deluge^18,19^. A typical experiment at the LCLS can produce dozens of terabytes of data per day, and the European XFEL is expected to raise this into the hundreds of terabytes or beyond. That is comparable to ATLAS and CMS experiments at CERN, yet the existing data processing infrastructure is clearly inferior to the arrangements at CERN. The facilities as well as their user communities face problems of storage, archiving and computational analysis of the data. The gap between the ability to produce and handle data is increasing. In other fields the standard approaches to this problem include lossy data compression, low­level trigger-based vetoing and real time data ­mining and data ­management methods for smart data organization and reduction. For X-ray experiments most of these solutions cannot be applied. It is often requested to capture ‘all data’. Because of the inverse nature of diffraction imaging, complex analytics must be applied to evaluate the usefulness of a specific shot before deciding whether to keep it or reject it. For all imaging techniques the smallest representation of Big Data is a final result, e.g., a reconstructed three-dimensional structure, or four-dimensional movie (like in time-resolved studies). Steps moving from collecting and saving individual noisy shots, towards saving only the data contributing to a model or a set of models will provide substantial data reductions. Development of methods for smart real­time classification and assessment of the streamed data are an effective remedy to most of the problems, while increasing the relative proportion of valuable data.

Data sets such as those available in this Collection provide invaluable test sets for refining real-time assessment strategies. Such strategies are becoming increasingly important as the fraction of data that can be stored decreases with the advent of superconducting accelerators and XFELs with megahertz repetition rates.

### CXIDB

The data are now available to the scientific community from the Coherent X-ray Imaging Data Bank (CXIDB)^19^, a worldwide data bank for ultra-fast diffractive imaging. Data banks with experimental data are crucial for education and research, aiding the development and validation of new theories and techniques. CXIDB is dedicated to the archival and sharing of data from experiments with free-electron lasers. Such data are currently available only to an extremely limited number of people. In terms of uniqueness, X-ray lasers are not unlike space telescopes; they open a new window on the world, but only a few of these instruments exist today and the infrastructures are heavily over-subscribed. CXIDB enables anyone to upload experimental data and browse data deposited by others. Entries can be downloaded from http://www.cxidb.org.

### Software

Publication of this Collection of Data Descriptors coincides with the publication of a special issue of the *Journal of Applied Crystallography* on software for research with free-electron lasers (http://journals.iucr.org/special_issues/2016/ccpfel/ and ref. 20). The software collection covers a range of topics such as simulation of experiments, online monitoring of data collection, diagnostics of hits and data quality, data management, phasing and analysis for both nanocrystallography and single particle diffractive imaging.

The two Collections represent the first salvo in the battle to bring under control the data torrent unleashed by new XFELs. Such a trove of tools should also prove most useful to any researcher wishing to analyse the data made available by the Collection of Data Descriptors in *Scientific Data* and deposited in the Coherent X-ray Imaging Data Bank^19^.

## Additional Information

**How to cite this article:** Maia, F. R. N. C. & Hajdu, J. The trickle before the torrent—diffraction data from X-ray lasers. *Sci. Data* 3:160059 doi: 10.1038/sdata.2016.59 (2016).
